# The Temporal Investigation of Multimodal Elements (TIME) Study: protocol for an observational, longitudinal study to characterize the dynamic structure of molecular and digital data in older adults in stable health

**DOI:** 10.3389/fdgth.2026.1881042

**Published:** 2026-10-21

**Authors:** James T. Yurkovich, Emma Glass, Audrey N. Nash, Noah Levine, Sophia Lee, Katheryn Ehlen, Eduardo Hernandez, Prabin Gharti, Chatura Senadheera, Ashen Fernando, Drew Witherington, Lance Pflieger, Jyotika Erram, Noa Rappaport, Alison Le, John C. Newman, Brianna J. Stubbs

**Affiliations:** 1 Buck Institute for Research on Aging, Novato, CA, United States; 2 Phenome Health, Seattle, WA, United States

**Keywords:** aging, digital health technologies, observational study, phenomic data, phenomics, predictive health, systems biology, temporal dynamics

## Abstract

**Background:**

Biological systems exhibit dynamic patterns over multiple temporal scales—from minutes to months—that are poorly captured by conventional cross-sectional or low-frequency longitudinal studies. These patterns, including diurnal variations and underlying continuous rhythms, may be critical determinants of health, resilience, and disease risk in aging. Existing longitudinal studies in older adults lack high-frequency, multimodal measurements that integrate molecular, physiological, and digital health data streams.

**Objectives:**

The TIME Study aims to: (i) Characterize temporal patterns in molecular, physiological, and digital health measures in healthy older adults; (ii) determine how these patterns vary across biological domains and relate to each other; and (iii) assess how physiological systems respond to defined perturbations (oral glucose tolerance and maximal exercise).

**Methods:**

TIME is a single-site, observational, longitudinal study enrolling up to 150 adults aged ≥55 years. Over an 11-week main phase, participants complete seven weekly low-frequency visits, two perturbation challenge visits, and two, two-day high-frequency sampling epochs. Biospecimens, clinical measures, cognitive and physical performance tests, and continuous digital health data are collected. Follow-up visits occur at 6 and 12 months.

**Expected impact:**

By integrating multimodal, temporally resolved data, TIME will provide a foundational dataset for understanding the role of biological rhythms in aging and inform future precision health strategies.

**Clinical Trial Registration:** https://clinicaltrials.gov/study/NCT07107386, identifier NCT07107386.

## 1 Introduction

The human body is a complex system that is governed by dynamic processes spanning different timescales. At the finest temporal scale, sub-second to minute-to-minute changes dictate neuronal firing and cardiac rhythms. Over hours, the body responds to environmental stimuli such as feeding and exercise through rapid metabolic shifts. On a daily and weekly basis, circadian rhythms and hormonal cycles regulate sleep architecture, basal physiology, and immune responses. Finally, over months and years, these continuous biological rhythms accumulate into the slow, gradual changes of aging. These interconnected processes are often studied in isolation, leading to a fragmented understanding of human physiology (1, 2). As biomedical technology rapidly develops, there is a critical need to understand how these innovations can be best applied to human health (3). One challenge is that many newer molecular profiling technologies (e.g., proteomics, metabolomics, lipidomics, methylation) have not been rigorously studied to understand how the time of day, fasting, or exercise impacts measurements (4–6) This lack of understanding creates a barrier to translating these technologies into clinical practice, where they could provide valuable insights into health and disease (3, 7).

Concurrently, the landscape of patient monitoring is being transformed by digital health technologies (DHTs), which offer a scalable solution to the limitations of traditional, episodic clinical assessments (8, 9). By enabling continuous, non-invasive data collection in real-world settings, wearables and mobile health platforms democratize access to high-frequency physiological tracking while reducing clinical costs and patient burden (10, 11). While these tools provide real-time resolution of biometric rhythms such as sleep patterns, activity, and resting heart rate, their full predictive utility is hindered by a disconnect from the patient's underlying biochemical state. This disconnect is not due to a lack of measurement capability, but rather a historical lack of paired, temporally synchronized data. While digital sensors and deep phenomic profiling have both advanced independently, these modalities are rarely captured simultaneously in the same individuals. Consequently, there remains a critical gap in rigorously integrating continuous digital health streams with deep biological measurements to holistically map the multi-scale temporal dynamics of human physiology (12).

Here, we present the Temporal Investigation of Multimodal Elements (TIME) study, an observational, longitudinal project designed to systematically map the temporal dynamics of the human phenome—the complete set of molecular and digital markers that define an individual's biological state—over one year. To achieve this, the study deliberately pairs continuous digital markers with high-resolution molecular profiles to capture an individual's synchronized biological state. The TIME study explicitly addresses both (i) the lack of paired overlap between digital and molecular datasets, and (ii) the longitudinal variation (e.g., time-of-day, week-to-week) inherent within the molecular data itself by rigorously capturing both modalities in tandem. The study's overall goal is to provide a comprehensive understanding of how the human body changes over time, providing a foundational dataset to informthe development of next-generation diagnostic tools and personalized health interventions. The TIME study's primary hypothesis is that diurnal variations and underlying continuous rhythms substantially impact molecular and physiological processes, thus directing the development of personalized and predictive health interventions. A key objective is to rigorously integrate continuous wearable metrics with high-resolution biochemical data, bridging the gap between digital phenotypes and their associated molecular signatures. The comprehensive temporal profiling provided here is designed to reveal novel insights into the dynamics of human physiology and result in a mapping of these complex temporal signatures. This knowledge will pave the way for a future where healthcare is proactive, predictive, and tailored to the unique rhythms of each individual.

## 2 Methods

### 2.1 Study design overview

TIME is a single-site, observational, longitudinal study designed to characterize temporal biological patterns in older adults in stable health. The study is primarily discovery-driven, recognizing that there is currently insufficient data to form specific hypotheses regarding time-of-day or week-to-week dependence for many omic endpoints (e.g., proteomics, metabolomics) in older adults.

Over a 2.5-year enrollment period, the TIME study will capture a wide range of time points and naturalistic conditions (Figure 1). Seasonal variation will occur and is not explicitly controlled for; instead, it is documented and treated as a feature of the dataset, providing an additional axis of variability to inform future targeted investigations.

**Figure 1:**
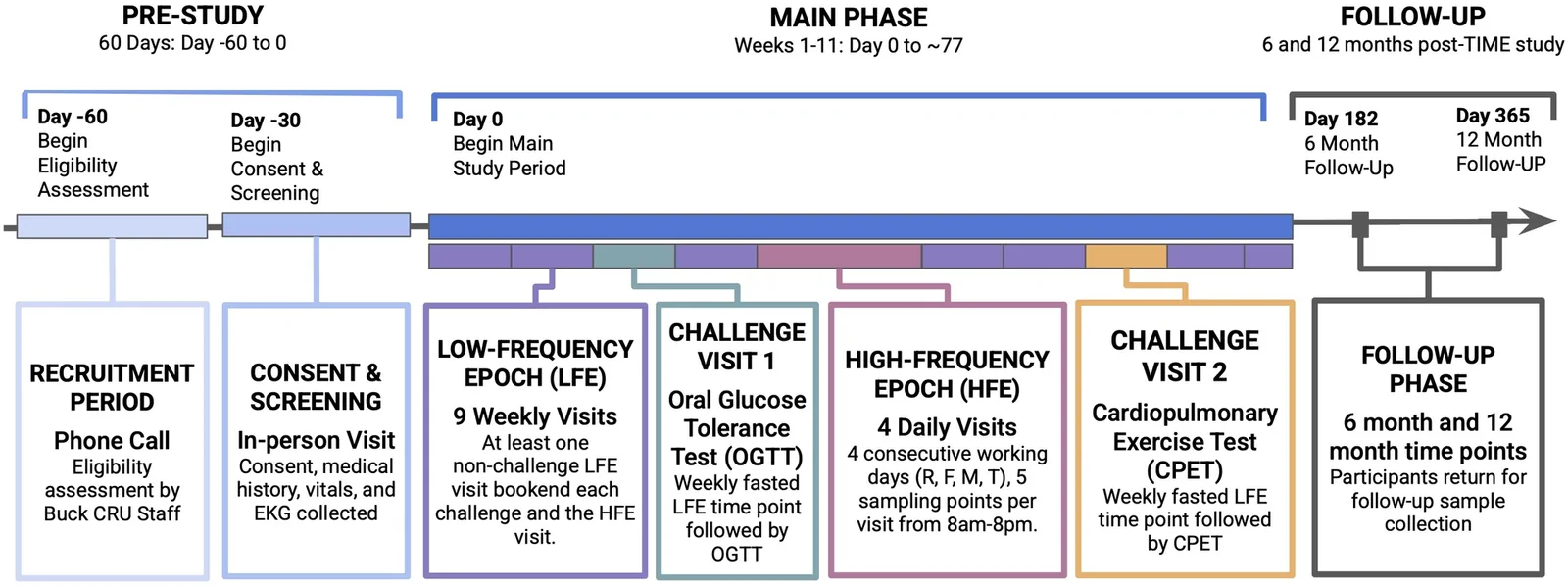
Overview of the TIME study. At each week's visit, vitals, blood, urine, cognitive testing, physical function testing, retinal scans, and facial imaging are collected, as well as skin, oral, vaginal, and stool microbiome samples. Oura Rings, WHOOP Bands, Stelo by Dexcom CGMs, and Withings Body Pro 2 scales are used throughout the study.

Participants will be studied in detail for the main 11-week period, and then a follow-up period will see the collection of biospecimens and measurements 6- and 12- months after enrollment (Figure 2). Digital health data will be collected throughout the main and follow-up periods. The main 11-week phase involves 9 total weekly visits: 7 standard low-frequency epoch (LFE, single fasted biospecimen collection and limited other measures), and 2 challenge visits (including the normal LFE sampling), and 4 days of high-frequency epoch (HFE, 12 h of regular biospecimen collection and comprehensive measures) on a consecutive Thursday, Friday, Monday and Tuesday. These timepoints were chosen to allow both week-to-week and intra-day assessment of biological variation. The choice to do HF sampling at the beginning and end of the working week rather than across a week allows us to examine the impact of changing behaviors such as sleep, diet, or exercise over the weekend. We also decided to capture data from two consecutive days, twice, so that each individual can act as their own biological control across days.

**Figure 2:**
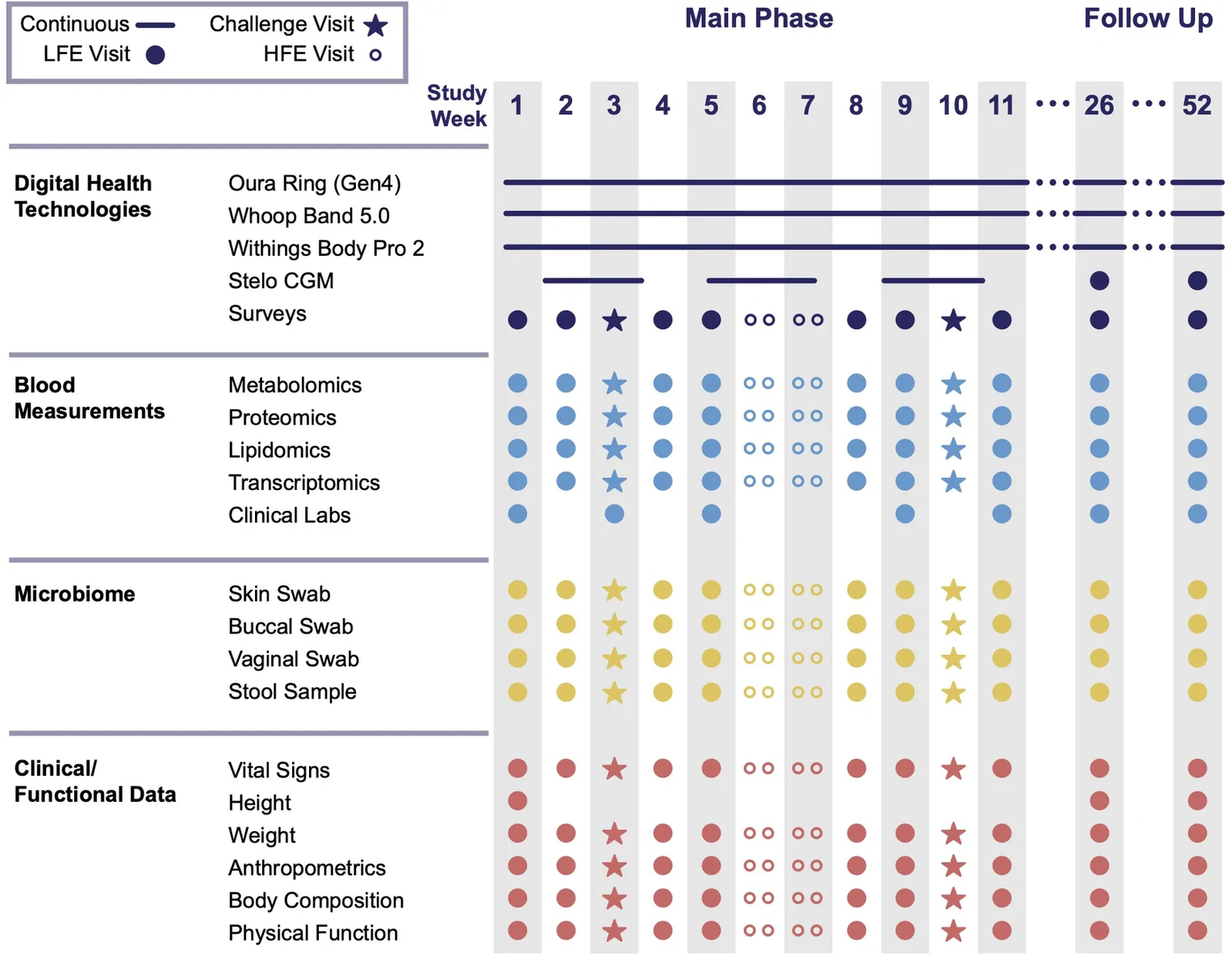
Overview of the TIME study data collection patterns. Schematic represents participant sampling and data acquisition for a subset of data modalities across study Main Phase and Follow Up timepoints (in weeks). Specific protocols include continuous digital monitoring (Oura Ring Gen4, Whoop band 5.0, Withings Body Pro 2), 14-day continuous glucose monitoring (CGM) windows, high-frequency epoch (HFE) collection windows (four days with multiple daily measurements across two weeks), weekly in-person visits, and challenge tests. Protocol guidelines allow for flexibility in scheduling individual visit types within each week (see Sec. 2.6.3 for details), meaning the challenge visits may not always occur during the same week for each study participant (see Sec. 2.6.3 for details). For a detailed look at data collection patterns during HFE visit days, see Figure 4. A full enumeration of DHTs deployed and all current data sources is in Supplementary Table S2.

On two of the weeks, participants will complete challenges designed to capture the response of the body to perturbations: an oral glucose tolerance test (OGTT), and a maximal cardiopulmonary exercise test (CPET). The inclusion of the OGTT and CPET allows for the mapping of the dynamic nature of the human phenome; biological systems operate across complex physiological timescales, and cross-sectional sampling often fails to capture underlying systemic behavior (13). Prior work has shown that dynamic metabotypes and molecular network properties revealed by a glucose challenge (14), or by a bout of exercise (15), are entirely unobservable in steady-state, resting profiles. By introducing the OGTT and CPET as acute, standardized perturbations, we capture transient molecular dynamics following food consumption and exertion that would otherwise be missed. The two challenges also probe deliberately orthogonal physiological axes—metabolic/endocrine and cardiopulmonary/autonomic—and follow internationally standardized protocols (WHO (16)/ADA criteria for OGTT; AHA (17) graded exercise testing criteria, using a braked bicycle ergometer, for CPET), allowing precise, reproducible administration and direct comparison to existing reference and epidemiological data. Both outcome measures are independently established predictors of long-term health risk, with 2-hour glucose tolerance during the OGTT predicting all-cause and cardiovascular mortality independent of fasting glucose (18), and VO_2_max recognized as one of the strongest predictors of all-cause mortality (19). While standard clinical assays have been extensively validated against confounds such as fasting and exercise, newer research-grade platforms (e.g., proteomics, metabolomics, lipidomics) have not undergone this level of validation; applying these same standardized perturbations to these platforms allows us to characterize how broad omic profiles respond to known cyclic and behavioral variables, generating a reference resource for correcting variability in future phenomic datasets. Finally, because a primary aim of TIME is to bridge near-continuous digital health data with discrete molecular data, the OGTT and CPET provide targeted, well-timed metabolic and physical stress events that can be directly correlated with concurrent data from continuous glucose monitors and wearable devices, allowing complex molecular signals to be mapped to accessible digital proxies that are otherwise invisible at baseline (20–22).

By capturing high-frequency (daily), low-frequency (weekly), challenge, and follow-up (6- and 12-month) data, TIME will create a foundational dataset that can be used to bridge the gap between discrete molecular measurements and near-continuous digital health data.

The study protocol described here has been approved by Advarra Institutional Review Board (current version 2.0 approved on June 25th 2026, IRB reference: Pro00085349), any further amendments will be approved by the IRB. Participants will also be informed of any protocol modifications affecting their study procedures and will provide updated consent (initials and date on the relevant ICF section) prior to undergoing any modified procedure. The study is registered with clincaltrials.gov (NCT07107386), all items from the WHO Trial Registration Dataset are included in Supplementary Table S1. Enrollment began on June 15th 2025 and is expected to run until June 2027. Here we present details of the study protocol according to SPIRIT 2025 guidelines (Supplementary Table S1).

### 2.2 Study setting

The TIME Study is conducted by investigators and clinical research staff at The Buck Institute for Research on Aging, a non-profit research institution based in Northern California, USA, in partnership with Phenome Health, a Seattle-based nonprofit. The majority of participants are expected to be residents of Northern California.

### 2.3 Study population

Participants in TIME (up to *n* = 150) will be adults at or over the age of 55 years in stable health. We aim to recruit an even number of male and female participants and a population representative of the demographics surrounding the clinical site. The choice to focus on adults over the age of 55 years was intended to reduce variability. Firstly, the human phenome is known to change across the lifespan (23), narrowing the enrollment window reduces natural variability occurring with age (24). Secondly, in females the menstrual cycle and the menopause transition are known to cause increased variability on both a micro and macro level (25, 26), and so recruiting a population without a menstrual cycle and likely to be post-menopause removes this potentially large source of variability. However, we strongly believe that a future study should apply the same dense phenomic sampling approach to younger adults, especially women ages 18–45 to examine changes across all phases of the menstrual cycle.

Eligibility criteria are intentionally broad to capture a range of health statuses representative of the older adult population, while ensuring safety for all study procedures. Stable chronic diseases are not automatically exclusionary unless judged uncontrolled or clinically active by the study medical officer. As this study involves frequent blood collection, participants with anemia, coagulopathies, recent blood donation, and poor venous access are excluded. Notably, participants must reside in the Pacific or Mountain time zone, may not undertake travel crossing more than two time zones during the main 11-week study, and may not be shift workers; these requirements aim to reduce possible effects of time zones on circadian biology. Similarly, we exclude participants using many sleep medications more than once per week in the three months prior to the initial visit. Finally, as we plan to collect many types of microbiome samples, we exclude any participant with antibiotic use within 3 months of the initial visit. Other medications and supplements are permitted if use has been stable for at least three months before the initial visits. These are recorded and may in fact be of interest during our multiomic analysis. If a participant is required to start a new medication, elects to start a new supplement, or has a new, non-life-threatening diagnosis during the study this is not exclusionary and will be recorded and examined during analysis. Full inclusion and exclusion criteria are found in Table 1.

**Table 1:** Inclusion/exclusion criteria.

Inclusion criteria
Subject is greater than or equal to 55 years of age, inclusive at Screening. Subject is willing and able to attend in-person visits at The Buck Institute (Novato, CA). Subject must reside in the Pacific or Mountain Time Zones. Subject is willing and able to comply with all study procedures. Able to speak, write and read English, given some study materials will be available only in English. Participants must have a personally owned smartphone capable of running study specific apps and use their phone for this study. This includes a willingness to download and use the study applications and sync their phone with the necessary study devices. Willingness to continuously wear all provided study wearable devices and permit researcher access to all data. Able to provide informed consent.
Exclusion criteria
Planned travel during the main 11-week study period that would necessitate missing multiple weekly visits or crossing more than two time zones (e.g., travel to the eastern time zone of the US) (self-report, screening visit). Subject is non-ambulatory (self-report, screening visit). Subject has been hospitalized overnight within 3 months of Screening (self-report, screening visit). Subject requires assistance with any activity of daily living, excluding continence (self-report, screening visit). Subject lives in an institutional setting (skilled nursing facility or residential care facility for the elderly) (self-report, screening visit). Subject works nights or shifts, or takes part in any activities that means it is not possible to maintain a consistent sleep-wake schedule during the main study (self-report, screening visit). Female participants must be post-menopause(self-report, screening visit). Musculoskeletal conditions that would prevent completion of the required exercise testing (self-report, screening visit). Pulmonary: Clinical diagnosis of any chronic pulmonary disease that prevents strenuous exercise (self-report, screening visit). Cardiovascular: presence of acute, uncontrolled or severe congestive heart failure, coronary artery disease, significant valvular disease, congenital heart disease, serious arrhythmia, pulmonary embolism, stroke, or symptomatic peripheral artery disease (self-report, screening test). Specific criteria used to determine whether a participant can undergo the screening CPET follow the American Heart Association (AHA) Criteria) (self-report, screening test, and clinician judgement). Uncontrolled hypertension: Blood pressure greater than 180 mm/Hg systolic or 100 mm/Hg diastolic is immediately exclusionary. Blood pressure greater than 150 mm/Hg systolic or 90 mm/Hg diastolic requires medical officer review and repeat measurements at home may be requested (screening visit). Abnormal bleeding or coagulopathy: History of a bleeding disorder, wound healing disorder or clotting abnormality (self-report, screening visit). Anemia: Presence or recent history of anemia requiring treatment (e.g., iron supplementation, blood transfusions, or erythropoiesis-stimulating agents) (self-report, screening visit). Blood donation: Whole blood, platelet or plasma donation in the last 3 months or plans for donation during the main 11-week study period (self-report, screening visit). Poor vein access: Participants with poor venous access per self-report, will be excluded to ensure successful and consistent blood collection procedures (self-report, screening visit). Unstable health: Subject has a presence of acute or uncontrolled and/or clinically active and/or advanced stage disease (self-report, screening visit). “Uncontrolled” and “clinically active” are per the judgement of the Medical Officer. Assessment should include the date of diagnosis and any recent active management, such as new medications or recent procedures relating to the condition. Stable chronic disease is not an exclusion criterion unless specified. Chronic disease that is managed with the patient's physician per shared decision-making, even if sub optimally by typically recommended care, is not an exclusion criterion unless uncontrolled or clinically active. Chronic antibiotic use: Use of antibiotics that is expected to be ongoing, regardless of frequency, throughout the main study period (self-report, screening visit). Psychiatric disorders: diagnosed psychiatric disorders that may impact study outcomes, including schizophrenia, bipolar disorder, schizoaffective disorder, or other severe mood or psychotic disorders at the discretion of the Medical Officer (self-report, screening visit) Subject has a condition the Medical Officer believes would interfere with their ability to provide informed consent, comply with the study protocol, which might confound the interpretation of the study results, or put the subject at undue risk
Excluded Medications
If a subject is taking any of the following medications within 3 months of the Screening Visit, they may not enroll into the study: Any antibiotic use. Sleep aids (used more than once per week): diphenhydramine, doxylamine, melatonin, ramelteon, benzodiazepines (e.g., estazolam, flurazepam, quazepam, temazepam, and triazolam), nonbenzodiazepine benzodiazepine receptor agonists (e.g., eszopiclone, zaleplon, and zolpidem), sedative antidepressants (e.g., trazodone, mirtazapine, doxepin), dual orexin receptor antagonists (e.g., Daridorexant, lemborexant, and suvorexant), quetiapine, or any other medications taken specifically for treating insomnia or sleep disorders per participant report Use of all other medications and supplements should be stable for the 3 months prior to Screening. “Stable” means a consistent pattern of medication and supplement use even if day-to-day use varies (e.g., constipation medications).

### 2.4 Study objective

The TIME study's primary objective is to comprehensively map human temporal dynamics by characterizing the dynamic interplay of molecular and digital health markers across daily and weekly timescales. This will be achieved through the analysis of high-frequency molecular data, including proteomics, metabolomics, and lipidomics, alongside continuous physiological data streams from wearable sensors. Despite the rapid innovation in biological measurement technologies, the field has not identified a minimum viable dataset required to accurately characterize an individual's unique, dynamic biological state. It remains undetermined which specific combinations of variables or datatypes—whether continuous digital phenotypes or discrete multi-omic signatures—serve as the most robust, actionable biomarkers. By systematically capturing these dense, multimodal data streams in tandem, the TIME study aims to construct a robust translational framework to investigate the complex correlations across diverse biological domains. This approach will allow us to evaluate the extent to which easily accessible, non-invasive wearable metrics reflect biochemical fluctuations. Ultimately, by mapping these connections, this study provides a highly valuable dataset to distill the vast human phenome down to a focused, practical dataset, highlighting candidate biological and digital biomarkers necessary to drive scalable precision health interventions.

### 2.5 Study data

Data collection will occur from a range of biological samples, digital devices, participant reported measures, and clinical assessments (Figure 2). There are, of course, a multitude of functional, clinical, digital, and molecular technologies that can be used to profile human physiology. We identified two major considerations when selecting the technologies to be used in the TIME Study:

(1) Data interoperability: To maximize the translational impact and utility of the TIME dataset, we prioritized analytical platforms that ensure seamless cross-compatibility with major, population-scale cohorts (27–32). By selecting established, industry-standard technologies—such as the Olink platform for global proteomics and Metabolon for global metabolomics—we deliberately align our molecular readouts with the foundational protocols employed by massive longitudinal initiatives like the UK Biobank (28) and the NIH *All of Us* Research Program (27). This strategic harmonization enables researchers to leverage vast external reference datasets to contextualize the high-frequency temporal dynamics and personalized biorhythms mapped within our study.
(2) Future-proofing: Recognizing the rapid evolution of multi-omic profiling and biomarker detection technologies, a core priority of the study design is the long-term future-proofing of our physical biorepository. We have instituted rigorous, standardized protocols for the high-volume collection, comprehensive processing, and long-term cryogenic banking of diverse blood fractions (e.g., plasma, serum, peripheral blood mononuclear cells). By establishing a high-quality physical archive alongside the immediate digital and molecular datasets, we ensure that as novel, higher-resolution analytical technologies inevitably emerge, these uniquely valuable, temporally dense longitudinal samples can be retrospectively analyzed (33). This biobanking strategy maximizes the scientific yield of the study and guarantees that the TIME cohort remains an enduring, adaptable resource for future discoveries in predictive health.

#### 2.5.1 Digital health technologies

DHTs serve as the foundation of the TIME study's longitudinal architecture, providing the continuous physiological context necessary to accurately interpret episodic clinical datatypes. By passively capturing a high-frequency stream of biometric and behavioral data in a natural, day-to-day setting, these tools establish a robust reference profile for each participant's baseline state and daily lifestyle. Crucially, the deployment of advanced multimodal sensors—such as continuous glucose monitors, smart scales, and wearable devices—links the study's low-frequency biological sampling time points (Figure 2). This dense digital phenotyping prevents the loss of vital dynamic information between physical clinic visits, allowing us to continuously monitor physiological strain, recovery, and biophysical fluctuations. Ultimately, anchoring deep, multi-omic molecular profiling to this continuous digital framework is what enables the accurate mapping of multi-scale temporal dynamics. A summary of the specific DHTs deployed, alongside all current data sources and expected endpoints, is detailed in Supplementary Table S2.

### 2.6 Participant timeline

#### 2.6.1 Recruitment

Participants will be recruited using advertisements via email, website banners, social media, flyers, community outreach and partnerships, and referrals. Recruitment materials will be designed to provide clear and concise information about the study, including its purpose, eligibility criteria, compensation, and contact information for interested individuals. Potential participants will be identified based on the study's inclusion and exclusion criteria and approached through in-person interactions at community and private events, phone calls, emails and mailed invitations.

Efforts will be made to ensure recruitment reflects the population of the San Francisco Bay Area, with targeted outreach to senior community centers, independent living communities, clubs, gyms, religious centers, and academic institutions to enhance the generalizability of study findings.

#### 2.6.2 Telephone screening

During the telephone screen, subjects will be provided with a summary of the study and assessed for initial eligibility.

#### 2.6.3 In-person visits

For all in-person visits, participants must meet the following pre-test criteria: fasting ≥10 h, no alcohol ≥10 h, no strenuous exercise ≥10 h, no psychoactive cannabis products ≥10 h. Participants will be queried on arrival to confirm compliance. At every visit, participants will be queried on their use of any new medications and supplement, or any changes to their health. Participants will also be queried to ensure compliance with study protocol, and will be reminded of study instructions.

As the main phase of the protocol is strenuous, requiring weekly in-person visits for 11 weeks, we allow some flexibility in timing to facilitate participant retention. One LFE in-person visit must occur per working week (Monday–Friday) during the 11-week main period; there will be an allowable window of minimum four days and a maximum of nine days between all study visits. Similarly, if nine visits cannot be completed within 11 weeks, it is allowable to take up to 15 weeks to complete all required visits. LFE visits do not take place during the two working weeks when HFE visits are scheduled. In addition, we plan to flexibly schedule the two challenge visits and the four-day high frequency sampling block. A final relevant retention strategy will be to pay stipends in a staged manner, increasing in value the further a participant progresses through the study.

There must be a non-challenge, LFE weekly visit before each of the challenge visits and before the HFE sampling block. The oral glucose tolerance challenge occurs first (between Week 2–5), followed by the HFE sampling block (Weeks 4–8) and lastly the maximal exercise test (Weeks 6–10).

Given the study's core focus on multi-scale temporal dynamics, all biospecimen collections will be strictly time-stamped upon acquisition, and strict adherence to standardized, time-gated processing windows—from initial draw to final preservation—will be enforced to eliminate pre-analytical temporal confounding. A study timeline in the SPIRIT Figure format is shown in the Supplementary Information.

#### 2.6.4 Screening visit (visit 1)

Staff will review the informed consent form with potential participants, provide time for questions, and confirm understanding before obtaining participant signature. Consent will be conducted in person in a private setting in accordance with Good Clinical Practice guidelines. The consent process will include options for broad data and biospecimen sharing for future research, as applicable. All study procedures are considered minimal risk. Blood draws, exercise testing, and glucose tolerance testing are standard procedures; adverse events are monitored per protocol.

Participants will provide written informed consent and a full medical history, medication and supplement use, demographic data, vital signs and a resting EKG will be obtained. These data will be reviewed by a study medical officer who will make final decisions on eligibility and enrollment into the study. No additional ancillary or post-trial care or compensation for research-related injury will be provided. Participants will be informed of this limitation during the informed consent process. Participants will be advised to seek medical care through their own healthcare providers in the event of any adverse health events arising during the study.

#### 2.6.5 Main phase (11 weeks)

##### 2.6.5.1 Low frequency visits (9 total weekly visits: 7 standard LFE visits and 2 challenge visits)

At a morning visit, a single LFE data collection time point will take place (∼0800H). This consists of a vital sign measurement, venous blood sample, a urine sample, a retinal scan, a saliva sample, a buccal swab, a skin swab, a vaginal swab (female subjects only), cognitive testing and grip strength testing. Subjects will be given at-home kits for collection of a stool sample within the 3 days prior of all LFE visits.

At the first and last LFE visits additional procedures will be performed. This will include anthropometric measures, a subset of the self-reported measures, resting metabolic rate measurement, and set-up and distribution of study devices (first LFE visit only). At the LFE visit prior to both challenge visits and the HFE visits, a continuous glucose monitor will be applied to the upper arm, and participants will be given standard meals to consume prior to the challenge or HFE visits.

##### 2.6.5.2 At-home procedures

Participants will be asked to wear and sync study wearable devices throughout the 12-month study, and to record their weight twice daily using the Smart Scale (Withings Body Pro 2). To incentivize participation and increase retention, participants who complete the main study period may keep the study devices. During the main 11 week study period participants will be asked provide to routine data tags about activities (e.g., exercising, napping) and other self-measurements (e.g., blood pressure) via SMS chatbot, additionally during the three two-week periods where a CGM is worn, participants will be asked to photograph all food consumed and share via SMS Chatbot. If a CGM fails or falls off before the 15-day window, it is replaced if feasible.

##### 2.6.5.3 SMS Chatbot

The current state of the art in behavior tracking is transitioning away from high-burden, app-based logging toward low-friction, conversational interfaces. By utilizing natural language processing and computer vision directly within native messaging platforms, modern ecological momentary assessment (EMA) tools significantly reduce recall bias and participant fatigue compared to traditional dietary and lifestyle diary (34–37). The SMS Chatbot (“Bucky”), a next-generation AI-backed system, is designed to support the TIME Study by collecting data in a natural, conversational manner. Its primary function is to engage with participants via SMS texting (i.e., no additional mobile application required) to collect additional data and information. This is achieved through two types of interactions: user-initiated messages, such as sending photos of food; and SMS Chatbot-initiated messages, which are scheduled to collect information at specific times, such as around meals or after a workout. These data are timestamped and correlated with other data collected from the participants' wearables.

The SMS Chatbot is governed by strict guardrails, ensuring the accuracy and completeness of its responses. Its functionality is intentionally limited to a predefined set of tasks, including wearable integration, daily habit tracking, food and nutrition logging, and user data retrieval. The knowledgebase is derived from a curated selection of peer-reviewed literature and books, and it is explicitly designed to not provide medical advice. To further ensure security and prevent manipulation, the SMS Chatbot undergoes rigorous daily testing. The goal of this system is to increase retention and engagement of study participants by answering questions and facilitating the collection of additional data from questionnaires, surveys, and daily occurrences.

##### 2.6.5.4 Study meals

Study participants will be consuming a total of 25 standardized study meals immediately before and during the challenges. During in-clinic HFE visits, meals are provided at approximately 0845, 1200, and 1800H. Meals are designed to align with a Mediterranean dietary pattern (10%–35% protein, 45%–65% carbohydrate, and 20%–35% total fat). Participants select from a menu of 11 pre-made options, with omnivore, gluten-free, and vegetarian selections available to accommodate a range of dietary preferences. Total daily energy expenditure (TDEE) is estimated for each participant using the Mifflin-St Jeor equation (38)

RMR=(10×Weightinkg)+(6.25×heightincm)−(5×ageinyears)+s

where s is +5 for males and −161 for females with a sedentary activity factor (1.2), reflecting the limited physical activity expected during in-clinic study days. Portions are scaled to meet 100% of TDEE and maintain an energy-neutral balance, with supplemental nutrition shake(s) provided alongside the study meals as needed. Meals are delivered frozen and reheated on site; for at-home meal periods, participants receive frozen take-home meals. Compliance with meal and shake consumption is assessed by photographing each item on a scale before and after eating to quantify intake by weight. During at-home meal periods, participants are free to consume their provided meals at times of their choosing. Samples of the meals will be saved for future molecular analysis (e.g., using mass spectrometry) to perform integrative analyses with corresponding molecular data being generated from biospecimens.

##### 2.6.5.5 Challenge visit: oral glucose tolerance test (between weeks 2–5)

At the start of the visit, an IV cannula will be inserted and the LFE fasting blood sample will be collected following completion of the other LFE measures, directly before the OGTT is initiated. Participants will be asked to consume a drink containing a standardized amount (75 g) of glucose (Glucola) within 5 min. Venous blood sample collection will occur at 10, 20, 30, 60, 90, 120 and 180 (±5) minutes after administration of the oral glucose bolus (Figure 3).

**Figure 3:**
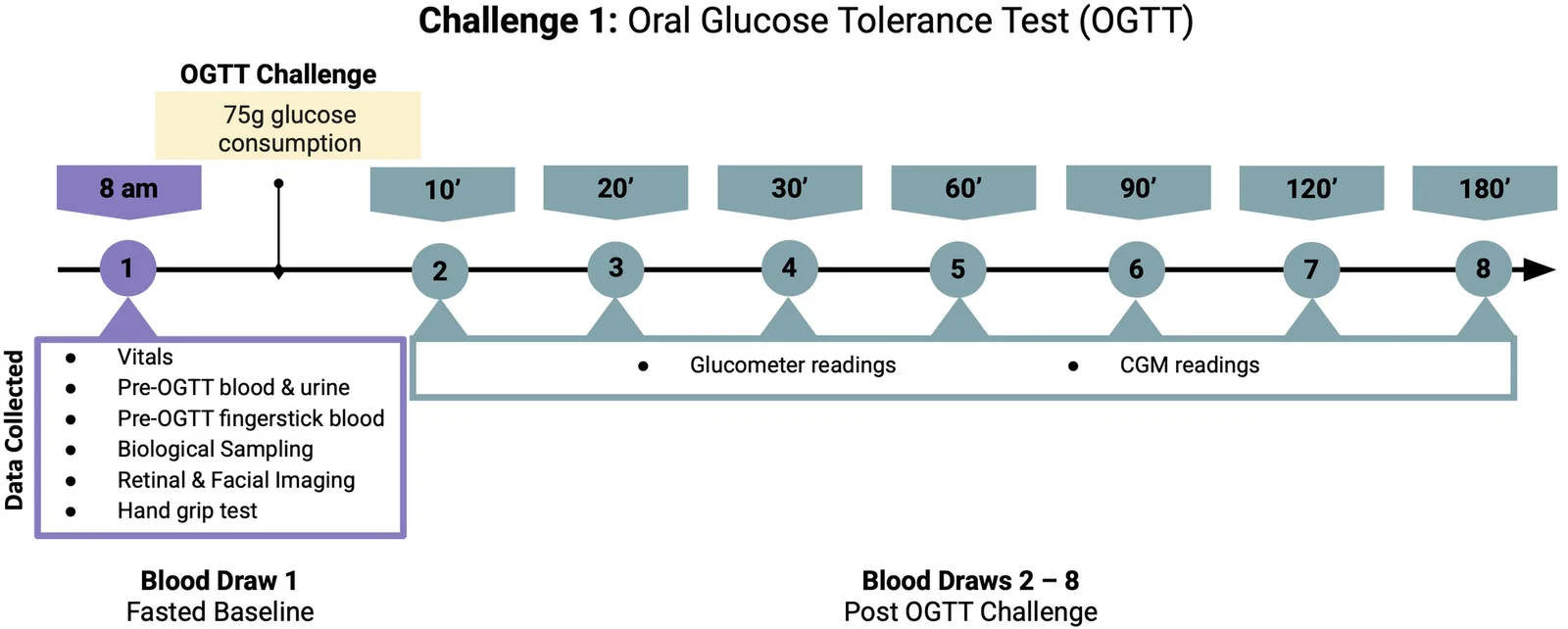
Challenge 1: oral glucose tolerance test (OGTT) sampling schedule.

##### 2.6.5.6 High frequency sampling block (between weeks 4–8)

Four near-identical in-person HFE visits occur over ∼12 h (0800–2000H) on a Thursday (Visit 1), Friday (Visit 2), Monday (Visit 3), Tuesday (Visit 4). Whilst we considered that this schedule might alter a participant's regular sleep-wake schedule, data from the wearable devices will allow us to account for the possible interaction between an individual's circadian timing and sample timing, and a set schedule mimics the nature of many other studies and clinical procedures that require fasting, as well as increasing operational feasibility for the clinical team.

Data will be collected at five time points: 0800, 1100, 1400, 1700 and 2000H. Data collected at each time point varies, but includes blood (from an IV cannula), urine, saliva, retinal imaging, facial imaging, cognitive function, grip strength, buccal swabs, vaginal swabs, a daily stool sample (using provided at home collection kit) and skin swabs. In the intervals between sampling time points, the following data will be collected: vital signs, anthropometrics, all study self-reported measures (measures will be completed once during the four visits, and will be split into batches to prevent fatigue), short physical performance battery, leg press testing, submaximal bicycle testing (Astrand-Rhyming test, also serves as familiarization for maximal exercise test) and spirometry (Figure 4).

**Figure 4:**
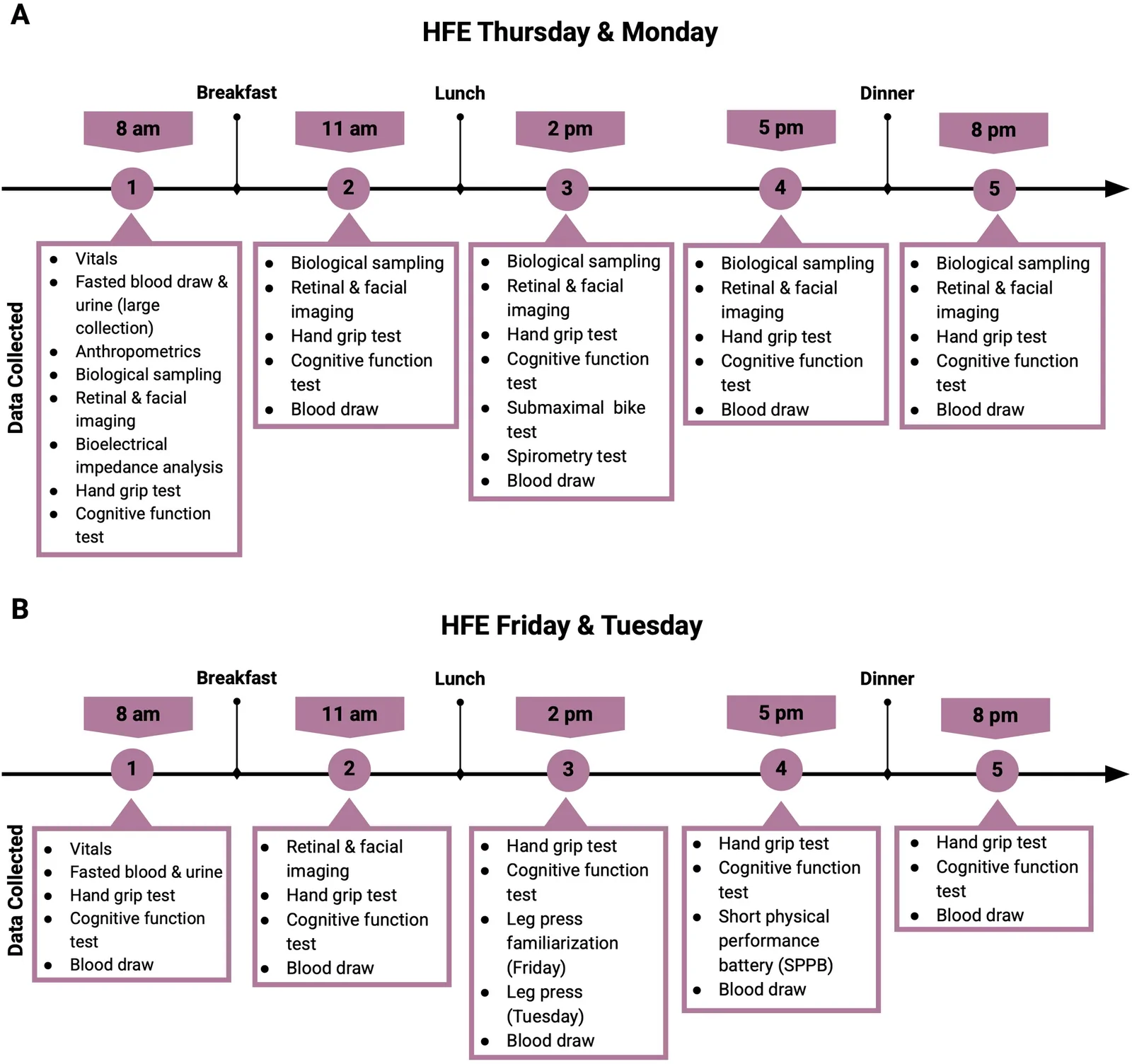
HFE sampling block. **(A)** HFE sampling schedule on Thursday and Monday. **(B)** HFE sampling schedule on Friday and Tuesday.

Three Study Meals will be provided at ∼0845H (immediately following the first data collection point), and during windows centered at 1200H and 1800H; or as desired by the participant. Photographs taken after each meal will be used to assess compliance and intake. Snacks, water, tea and coffee will be provided *ad libitum*, and all items consumed, and time of consumption will be recorded. Subjects will be asked to remain in the clinic and limit ambulatory activity.

At home between HFE visits, subjects will collect saliva samples before bed and on waking and collect a pooled overnight urine sample (Thursday and Monday). All meals over the weekend between the HFE visits will be provided by the study team and the following recommendations will be made for the weekend days between HFE visits: follow their usual weekend exercise and social patterns, and avoid extreme deviations (e.g., exercise longer or harder than usual, social gatherings that lead to extreme change their alcohol consumption or sleep schedule). Data will be captured over the weekend from the study wearables, allowing for an accounting of the effects of behaviors on biomarker data.

##### 2.6.5.7 Challenge visit: maximal exercise test (between weeks 6–10)

At the start of the visit, an IV cannula will be inserted and the LFE fasted blood sample will be collected following completion of other LFE measures. Then a standard meal will be provided and participants will rest for at least 1 h before the exercise test. Immediately prior to the test, EKG leads are placed for live reading during the test. Maximal aerobic capacity will be assessed via a graded exercise test to volitional exhaustion on a Lode cycle ergometer. Participants will wear a mouthpiece with nose clip for continuous breath-by-breath gas analysis (ParvoMedics TrueOne 2400). Workload will begin at 5–50 W and increase by 3–30 W·min^−1^ according to fitness level, aiming for test duration of 8–12 min after warm-up. Continuous measurements include oxygen uptake (VO_2_), carbon dioxide production (VCO_2_), minute ventilation (VE), respiratory exchange ratio (RER), heart rate (ECG or heart rate strap), and blood pressure at 2 min intervals. Perceived exertion is recorded using the Borg scal (39). Tests are terminated upon volitional fatigue, inability to maintain cadence, or meeting standard clinical termination criteria. Maximal effort will be confirmed by RER ≥1.10, heart rate within 10 bpm of age-predicted maximum, and/or a VO_2_ plateau (<0.2 L·min^−1^ change over final 2 min). Venous blood samples will be collected at 0, 15, 30, 60, 90 and 120 (±5) minutes after the end of the test, and cognitive testing occurs and 30 min post-test (Figure 5).

**Figure 5:**
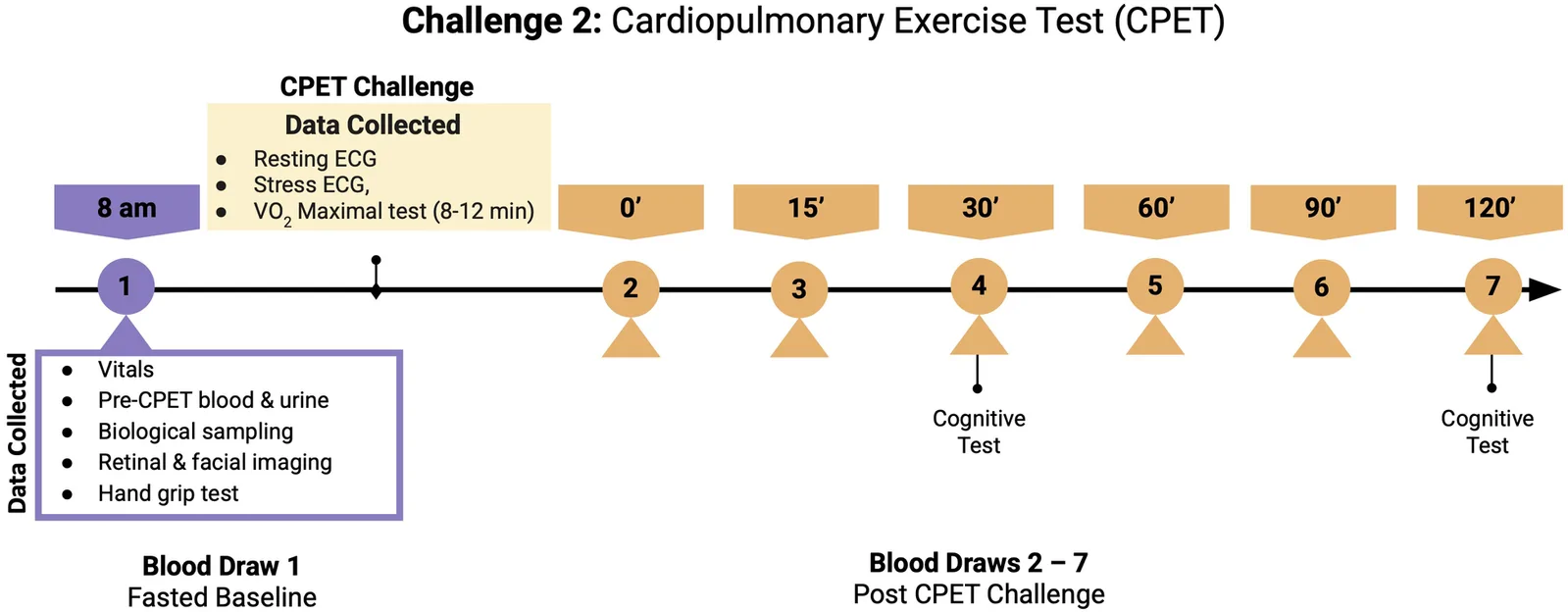
Challenge 2: cardiopulmonary exercise test (CPET) sampling schedule.

#### 2.6.6 Follow-up phase (6 and 12 months)

Follow-up in person visits replicate the structure of the Main Phase LFE visits. Vital signs and basic anthropometric data will be captured, along with blood for multi-omics analysis and clinical labs will be collected. Biological samples (stool, blood, urine, saliva, buccal swab, skin swab, vaginal swab) will be collected, grip strength will be tested, a retinal scan and facial images captured, and participants will complete a subset of study self reported measures and a subset of the cognitive tests.

### 2.7 Limitations, risks and safety monitoring

The chosen study design introduces several documented and controlled constraints. Specifically, the protocol does not implement systematic control for seasonal variation across the recruitment and sampling periods. This is mitigated by the comprehensive documentation of all sampling dates and times, which supports *post-hoc* statistical modeling to characterize and adjust for these effects (40). Furthermore, the study utilizes single-site, regional recruitment spanning the Pacific and Mountain time zones in the USA. While this approach simplifies multi-site data harmonization, it limits the immediate generalizability of the findings to the specific recruited population. As the study has an extremely high participant burden due to intensive visit schedules, the population who consent to participate will unavoidably select for currently unknown lifestyle or background characteristics, although we manage this through flexible scheduling and enhanced engagement strategies. Additionally, the intense monitoring required for the study introduces a risk of measurement reactivity, potentially altering the naturalistic behaviors we aim to observe. While this observer effect is unavoidable in such high-frequency phenotyping, we anticipate the study's duration will facilitate participant habituation, allowing *post-hoc* analyses to account for initial behavioral shifts.

Beyond these design limitations, several study procedures carry risks to participants, addressed through the following safety monitoring measures. This study was designated minimal risk by Advarra IRB (an OHRP- and FDA-registered independent IRB), and a Data Monitoring Committee was not required. In its place, the study's Medical Officers provide ongoing safety oversight, including eligibility review, adverse event adjudication, and real-time clinical consultation. Adverse events are graded for severity and relatedness to study procedures and captured via a standardized report form; serious adverse events are reported to the Institutional Official immediately and to the IRB per its required timeframe. Consistent with standard practice for minimal-risk, non-therapeutic research, no compensation is provided for research-related injury; participants are instructed to seek emergency care as needed and to contact the study team for non-emergency concerns.

The two procedures carrying the greatest physical risk—blood collection and the maximal exercise test—include additional targeted safeguards. Maximum single-day blood volume is 92 mL, with a total of ∼570 mL over the 11-week intensive phase (∼660 mL including follow-up visits; see Supplementary Table S3). An indwelling intravenous cannula avoids repeated venipuncture, hydration is confirmed via urine specific gravity prior to the OGTT, HFE visits, and CPET, and participants with baseline anemia are excluded at screening; a complete blood count is repeated at multiple points across the study, providing ongoing surveillance for treatment-emergent anemia. The maximal exercise test is conducted under continuous ECG and blood pressure monitoring by a physician and staff trained in advanced life support, with pre-test screening against AHA contraindication criteria and standardized termination criteria (17).

Several study procedures are also in clinical use and may yield findings of potential clinical significance. Such findings are reviewed by the study Medical Officer and, if confirmed relevant, disclosed to the participant for referral to their personal physician, per the Buck Institute's Incidental Findings SOP. Given the research-grade, non-CLIA-validated nature of the whole genome sequencing performed in this study, individual genetic results are not returned to participants.

## 3 Statistical analysis

### 3.1 Power calculation

The target enrollment for the TIME study is up to n=150 participants, with an anticipated completion cohort of n=125 allowing for potential attrition. For a discovery-oriented longitudinal protocol of this density, this enrollment represents a pragmatic, feasibility-driven target designed to optimize high-resolution multi-omic and digital phenotyping within operational and resource parameters. Statistical precision in this protocol is primarily driven by dense, repeated intra-individual sampling rather than cross-sectional sample size. The dense repeated-measure design provides greater statistical precision for intra-individual temporal trajectories, evaluating return-to-baseline kinetics following perturbation challenges, and quantifying normal biological variation.

The study recruits a community-dwelling cohort of adults aged 55 years and older, capturing a spectrum of baseline health statuses, comorbid conditions, medication regimens, and supplement uses typical of an aging population. Within-person temporal modeling and main cohort trajectories are the primary intended inferential objectives supported by the repeated-measures design. The study is not powered for definitive cross-sectional subgroup comparisons across specific age, disease, or medication strata. Consequently, all cross-sectional subgroup stratifications, multi-modal machine learning predictions, and secondary biomarker discoveries are explicitly designated as exploratory and hypothesis-generating.

### 3.2 Analysis plan

The statistical analysis plan for the study is designed to characterize the dynamic interplay within the human phenome by integrating disparate data modalities spanning molecular profiles, near-continuous digital streams, clinical metrics, environmental contexts, and participant-reported labels. To provide clear protocol structure while accommodating high-dimensional multi-modal discovery, analytical workflows are partitioned into a primary prespecified confirmatory framework and secondary exploratory pipelines.

All raw data will first be ingested into a centralized cloud repository built on a unified knowledge graph schema. To mitigate technical batch effects prior to data acquisition, biospecimen processing and assay runs enforce proactive experimental design controls, including sample randomization across assay plates with respect to participant identity and sampling visit, alongside the inclusion of repeated pooled quality control reference samples on every plate to monitor longitudinal run drift. Post-acquisition technical batch effects will be adjusted analytically using bridge-sample normalization. Missing data will be categorized and managed according to their underlying technical and biological mechanisms: continuous wearable gaps and sparse missingness in longitudinal visits will be handled through mixed-effects likelihood estimation, matrix completion, or Kalman filtering; uncollected visits or assay failures will be addressed using multiple imputation; and biological measurements falling below analytical limits of detection will be managed via left-censored single or multiple imputation procedures. Descriptive statistics will then be computed to summarize the data and identify preliminary patterns.

To separate short-term intra-individual dynamics from baseline inter-individual differences, primary statistical models will utilize linear mixed-effects frameworks. Time will be parameterized primarily as continuous calendar time (days elapsed since enrollment) to evaluate longitudinal drift across sampling visits, alongside secondary parameterizations of clock time (hours) to adjust for daily or weekly variation, and tertiary parameterization as age. Primary linear mixed effects architectures will fit participant-specific random intercepts to capture baseline variance alongside participant-specific random slopes to model individual-specific rates of temporal change. Multiple hypothesis testing across high-dimensional feature spaces will be controlled using Benjamini-Hochberg false discovery rate adjustments or permutation testing.

Acute dynamic responses during physiological challenges (OGTT and CPET) will be quantified using three primary scalar metrics derived from time-series curves: baseline-adjusted peak amplitude, net area under the curve above baseline, and recovery time (defined as the duration required to return within one standard deviation of pre-challenge baseline levels). Inter-individual differences in these response metrics will be evaluated using LME models adjusted for baseline covariates (biological sex, age, body composition). To complement these discrete summary metrics, full perturbation trajectories will additionally be evaluated using functional principle component analysis to decompose continuous response curves into orthogonal dominant modes of variation.

Rather than committing to a single rigid model architecture for complex discovery, a variety of dynamical systems analysis methods, such as time-scale decomposition, spectral analysis, and wavelet analysis, will be evaluated based on the observed sampling frequencies and signal distributions across data modalities. To bridge discrete molecular assays with high-frequency digital health streams, we aim to complement this approach with time-lagged cross-correlations, autoregressive modeling, and state-transition detection to map signals and characterize perturbation responses (41). Further modeling will aim to identify relationships and predict future states through both supervised and unsupervised machine learning (including time-series architectures and regularized regression), employing participant-stratified cross-validation to prevent data leakage and ensure model generalizability.

In the future, we hope to analyze the samples from study meals to investigate research questions relating to precision nutrition. Such analyses would link the molecular profile of consumed food (the input) to the subsequent molecular changes in human biospecimens (the output). This framework would involve a three-step process (42). First, input profiling captures a complete molecular picture of the dietary input using a multi-omic approach on both single ingredients and the final, prepared meal. Second, output profiling measures the human biological response by analyzing the molecular composition of biospecimens (e.g., blood, urine, stool). This step includes establishing a preprandial baseline in a fasted state, followed by collecting postprandial samples at multiple time points to capture the dynamic processes of digestion, absorption, and metabolism. Finally, data integration and biomarker discovery would link the input and output data to identify food-specific molecular signatures that reliably appear in biofluids post-consumption.

## 4 Data management and dissemination

Data will be entered into a secure, web-based electronic case report form (eCRF, RedCap) during study visits by study staff. Each participant will be assigned a unique study ID, and all identifying information will be stored separately from study data. Data will be checked for completeness and range validity, and pipelines will address missing data, outliers, and batch effects to ensure data quality. Access to study data will be restricted to the Principal Investigator and authorized study personnel, and all data will be stored on encrypted servers at The Buck Institute. Every effort will be made to maintain subject confidentiality before, during, and after the study by implementing a coding system and removing identifiable information. De-identified data will be made available upon reasonable written request to the Data Governance Committee for the TIME Study. However, recognizing that whole genome sequencing (WGS) and facial imaging carry inherent re-identification risks, these specific modalities will require a formal Data Use Agreement (DUA) and a Human Subjects Research Determination with documented approval from the requesting investigator's Institutional Review Board (IRB) if required. The DUA will explicitly prohibit any attempts to re-identify participants.

Where possible, we aim to share data with participants to incentivize retention. Participants can access their own wearable data throughout the study, and will be provided with a summary report at the end of the main phase. We will share hard copies of participant's clinical laboratory results and results of the body composition testing and the maximal exercise testing. As the multiomic data are not actionable or clinically validated, we do not plan to share this data.

We are committed to the sharing of final research data, being mindful that the rights and privacy of people who participate in research must be protected at all times and that there is the need to protect patentable and other proprietary data. All datasets will be free of any identifiers that would permit linkages to individual research participants and variables that could lead to deductive disclosure of individual participants. Detailed data management procedures, including SOPs for data entry, coding, security, and storage, are available upon request. Anonymized raw, subject-level data will be held at The Buck Institute under the custody of the Principal Investigator and de-identified data will be made available upon reasonable written request to the Principal Investigator. The results of the study will be disseminated via publication in scientific papers, abstracts, and presentations. Study results will be posted in trial registries (e.g., WHO, ClinicalTrials.gov) and summarized in plain-language summaries accessible to the public. Authorship will be determined by ICMJE guidelines, manuscripts will be prepared by the study team.

## 5 Discussion

Characterizing the temporal dynamics of a living system is crucial to understanding how the system works. Human biology is incredibly complex, with many processes operating at different timescales. Here, we describe a study targeted at quantifying these temporal dynamics in humans across data modalities ranging from DHTs to blood proteins and metabolites to the gut and vaginal microbiomes and functional measures—the TIME Study. Specifically, the TIME study is explicitly designed to address a dual gap in current biomedical research: (i) mapping the intrinsic temporal variation (e.g., time-of-day, day-of-week effects) within high-resolution molecular profiles, and (ii) establishing the direct overlap between these molecular rhythms and continuous digital health metrics. The design of the TIME Study has several important implications for human research.

First, TIME will serve as a reference dataset that quantifies biological variability across both daily (e.g., time-of-day, diurnal) and weekly timescales in older adults of stable health. The structure intentionally mirrors real-world longitudinal multi-omics studies to ensure relevance and scalability, including allowance for naturalistic variation in sampling times and seasonal effects. We anticipate that the resulting dataset will facilitate the development of novel statistical models for the robust characterization and adjustment of time-of-day and day-of-week effects on thousands of biological features. This foundational data—comprising on the order of 10,000 unique biological and digital signals at each time point—will directly inform the optimization of future longitudinal study designs by empirically defining the necessary temporal resolution and sampling frequency required to capture specific, highly dynamic biological signals.

Second, the inclusion of controlled physiological perturbations (an OGTT and a CPET) allows for the study of these same signals in response to acute metabolic and physiologic stress, and is critical for resolving the dynamic regulatory capacity of the biological system. Studying this stress response is akin to assessing a car's engine under load (the perturbation) rather than merely observing it while idling (the baseline state). The engine's performance metrics (molecular and physiological outputs) following a rapid acceleration or hill climb (the challenge) reveal its true functional limits and efficiency—specifically, how quickly and effectively the biological machinery returns to a stable state (homeostasis). These perturbations are necessary to study the system's resilience and adaptive speed, providing a far deeper measure of health than static baseline monitoring. Future iterations could incorporate additional perturbations targeting physiological axes not directly probed here. A psychosocial or autonomic stress challenge (e.g., a cold pressor test or Trier Social Stress Test) would engage the hypothalamic-pituitary-adrenal axis and sympathetic nervous system, both of which are only indirectly captured by the current metabolic and cardiopulmonary challenges. Given the study's focus on temporal biorhythms, a controlled sleep restriction or circadian misalignment challenge would be a natural extension, directly probing the chronobiological systems the study is otherwise designed to observe passively. A postprandial mixed-meal tolerance test could also complement the OGTT by capturing lipemic and broader hormonal responses to a more ecologically realistic dietary challenge than a pure glucose load. Each of these represents a feasible, previously validated perturbation that could extend the range of physiological systems captured in future TIME-like designs, at incremental cost to participant burden.

Third, the dense temporal sampling in the TIME study provides an empirical foundation to explore the concept of a Minimum Viable Dataset (MVD), defined here as a resource-optimized subset of measurements and sampling frequencies required to capture primary phenomic variation. Rather than establishing a universal standard, we aim to evaluate how MVDs might be derived analytically by applying feature-selection and utilizing temporal downsampling simulations (systematically reducing sampling intervals to observe signal decay and loss of predictive power). Such frameworks could help inform future study designs, assist target validation in drug discovery by accounting for temporal variance, or guiding clinical trial sampling schemes to reduce costs and participant burden while retaining statistical power While different applications will undoubtedly have different MVD requirements (e.g., a study investigating Alzheimer's Disease will likely need to measure different biomarkers than a study investigating Type 2 Diabetes), TIME has the potential to provide valuable insights into the fundamental temporal aspects of when to be measuring certain molecular and digital quantities. To enable independent teams to explore, test, and adapt MVD parameters for diverse clinical questions, dataset access will be available as outlined above.

Ultimately, the successful execution of the TIME Study will establish a critical baseline reference for the dynamic human phenome. It will provide insights into the significant operational logistics required to temporally profile the human system, lowering the activation energy required to run future studies on other cohorts that might study important biological patterns such as the female menstrual cycle. Finally, it will help define a MVD, centered around the rigorous integration of continuous DHTs with episodic specimen-derived biomarkers, that can inform the design of future biomedical studies. Once complete, the TIME resource stands to significantly advance our capacity to investigate the crucial role of time in homeostasis, health, and age-related decline by bridging the gap between digital phenotypes and underlying molecular associations.

The study protocol and any subsequent amendments were reviewed and approved by the Advarra Institutional Review Board (IRB reference:Pro00085349), 6100 Merriweather Dr., Suite 600, Columbia, MD 21044, call toll free; 877-992-4724). The TIME study is registered with ClinicalTrials.gov (NCT07107386, https://clinicaltrials.gov/study/NCT07107386). Trial registration date: 2025-08-06. All participants provided written informed consent prior to enrollment and participation in any study procedures.
