# Supplementary material for: The Temporal Investigation of Multimodal Elements (TIME) Study: protocol for an observational, longitudinal study to characterize the dynamic structure of molecular and digital data in older adults in stable health
Source: Front Digit Health. 2026 Oct 21;8:1881042. doi: 10.3389/fdgth.2026.1881042 (PMC13731243; doi:10.3389/fdgth.2026.1881042)
Supplement: Supplementary file 2 [file Table2.docx]

Supplemental Table 2 | A summary of the specific DHTs deployed, alongside all current data sources and expected endpoints

| **Data Source** | **Expected Endpoints** |
| --- | --- |
| **Devices and Digital Health Technologies** | |
| Continuous Glucose Monitor (Dexcom) | 24h - blood glucose  Glucose metabolism |
| Ring-wearable (Oura Ring Gen4) | Activity  Sleep  **Physiological measures**: heart rate, heart rate variability, temperature, blood oxygen saturation |
| Smart Scale (Withings Body Pro 2) | Morning and evening body composition and weight  Body composition changes |
| Wrist wearable (WHOOP Band 5.0) | Activity  Sleep  **Physiological measures**: heart rate, heart rate variability, temperature, blood oxygen saturation  Physiological strain and recovery |
| Facial imaging (XXX) | Facial age-score |
| Retinal imagine (TopCon NW500) | Retinal age |
| Patient Reported Outcomes | All of Us Research Program Basics Survey  All of Us Research Program Lifestyle Survey  All of Us Research Program Overall Health Survey  All of Us Research Program Personal and Family Health History Survey  All of Us Research Program Health Care Access and Utilization Survey  All of Us Research Program Social Determinants of Health Survey  All of Us Research Program Emotional Health History and Well-Being (Mental Health and Well-Being Survey 1)  All of Us Research Program Behavioral Health and Personality (Mental Health and Well-Being Survey 2)[^28^](https://www.zotero.org/google-docs/?1EpKMC)  Pittsburgh Sleep Quality Index (PSQI)[^29^](https://www.zotero.org/google-docs/?vfnKPf)  Patient Health Questionnaire 9[^30^](https://www.zotero.org/google-docs/?Un38Ur)  General Anxiety Disorder 7 Item (GAD 7) Scale[^31^](https://www.zotero.org/google-docs/?kj86u1)  Perceived Stress Scale-10[^32^](https://www.zotero.org/google-docs/?Pz9bxe)  Mindful Attention Awareness Scale[^33^](https://www.zotero.org/google-docs/?kKXliw)  Pittsburgh Fatigability Scale[^34^](https://www.zotero.org/google-docs/?fwVfZn)  Global Physical Activity Questionnaire (GPAQ)[^35^](https://www.zotero.org/google-docs/?GYQafX)  The Women's Health Questionnaire (WHQ)[^36^](https://www.zotero.org/google-docs/?ff6skM)  Munich Chronotype Questionnaire (MCTQ) DHQ III[^37^](https://www.zotero.org/google-docs/?qFfNUY) |
| Cognitive Testing (Posit, BrainHQ) | Dynamics of cognition  Domains include: processing speed, short-term memory, delayed memory, and executive function |
| SMS Chatbot | Food images  Study information |
| **Blood** | |
| Plasma | **Clinical labs** (includes but not limited to): Complete Metabolic Panel, Gamma glutamyl transferase, Complete Blood Count, Ferritin, Folic Acid, Heavy Metals Profile (including lead, mercury, cadmium, arsenic), Hemoglobin A1C, Homocysteine (Plasma), hs-CRP, Immunoreactive Insulin, Lipid Panel including, total cholesterol, triglycerides, HDL, ApoB, and calculated LDL according to the Friedewald equatio[^38^](https://www.zotero.org/google-docs/?Vbc06P), Lp(a), Methyllonic Acid, OmegaCheck(TM)), Thyroid Stimulating Hormone, Vitamin D 25-OH, glucose, C- peptide  Metabolomics  Proteomics  Lipidomics  Immune profile  Antibody Scan  Biobanking |
| Red blood cells | Banked for future research |
| Peripheral blood mononuclear cells | Transcriptomics  Banked for future research |
| **Other Biological Specimens** | |
| Saliva | Banked for future analysis  ProSpectral spectroscopy analysis |
| Urine | Metabolomics  Banked for future analysis |
| Buccal swab | Whole genome sequencing  Microbiome metagenomics |
| Skin swab | Microbiome metagenomics  Banked for future analysis |
| Vaginal swab | Microbiome metagenomics  Banked for future analysis |
| Stool sample | Microbiome metagenomics  Shotgun Metagenomic sequencing |
| **Clinical and Functional Data** | |
| Anthropometric data | Height  Weight  Body composition (bioelectrical impedance, RLJ Segmental System)  Waist, hip and upper arm circumference |
| Vital signs | Seated and standing blood pressure  Heart rate  Blood oxygen saturation  Temperature |
| Physical function | Hand grip strength  1-rep maximum leg press test  Leg press fatigability  Estimated VO2 max (Astrand-Rhyming test on Lode bicycle ergometer)  Direct measurement of VO2 max (incremental Lode bicycle ergometer test, Parvo metabolic cart)  Spirometry - lung function  Direct measurement of resting metabolic rate (Parvo metabolic cart)  Short physical performance battery (chair sits, 4m gait speed, balance testing) |
