# Supplementary material for: The Temporal Investigation of Multimodal Elements (TIME) Study: protocol for an observational, longitudinal study to characterize the dynamic structure of molecular and digital data in older adults in stable health
Source: Front Digit Health. 2026 Oct 21;8:1881042. doi: 10.3389/fdgth.2026.1881042 (PMC13731243; doi:10.3389/fdgth.2026.1881042)
Supplement: Supplementary file 3 [file Table3.docx]

**Supplemental Table 3 |** Blood volumes collected at each of the study visits.

|  | **First Non-C LFE** | **Non-C LFE** | ***LFE + OGTT** | ***HFE Visit 1 Thurs** | ***HFE Visit 2 Fri** | ***HFE Visit 3 Mon** | ***HFE Visit 4 Tues** | **LFE pre MaxE** | ***LFE + MaxE** | **Final LFE** | **6**  **Month** | **12**  **Month** |
| --- | --- | --- | --- | --- | --- | --- | --- | --- | --- | --- | --- | --- |
| **Number of specified visit type** | 1 | 5 | 1 | 1 | 1 | 1 | 1 | 1 | 1 | 1 | 1 | 1 |
| **Number of blood draws*** | 1 | 1 | 8 | 5 | 5 | 5 | 5 | 1 | 7 | 1 | 1 | 1 |
| **First draw volume (mL)** | 46.5 | 8 | 13 | 46.5 | 8 | 46.5 | 8 | 46.5 | 8 | 46.5 | 46.5 | 46.5 |
| **Volume - subsequent draws (mL)** |  | - | Samples 2 & 3  = 7 mL  Samples 4 - 8  = 13 mL | 8 | 8 | 8 | 8 | - | 8 | - | - | - |
| **Total Visit Volume (mL)** | **46.5** | **8** | **92** | **78.5** | **40** | **78.5** | **40** | **46.5** | **56** | **46.5** | **46.5** | **46.5** |
| **Total 11 week study volume (mL)** | **564.5** | | | | | | | | | | | |
| **Total 12 Month Study Volume (mL)** | **657.5** | | | | | | | | | | | |

* For all visits with multiple blood draws an intravenous cannula will be inserted to avoid repeated venipuncture. **Abbreviations:** HFE = high frequency epoch, LFE = low frequency epoch, NonC= non-challenge, CPET = cardio pulmonary exercise test, OGTT = oral glucose tolerance test.
